# Oral Health Attitude with the Socioeconomic Conditions of Mothers with Growth Stunting Children

**DOI:** 10.1055/s-0042-1757213

**Published:** 2022-10-28

**Authors:** Nadhira Aulia Putri, Asty Samiaty Setiawan, Arlette Suzy Setiawan

**Affiliations:** 1 Dentistry Bachelor Program, Faculty of Dentistry, Universitas Padjadjaran, Bandung, Indonesia; 2 Department of Community Dentistry, Faculty of Dentistry, Universitas Padjadjaran, Bandung, Indonesia; 3 Department of Pediatric Dentistry, Faculty of Dentistry, Universitas Padjadjaran, Bandung, Indonesia

**Keywords:** socioeconomic, mothers' attitude, stunting

## Abstract

**Objective** This study analyzes the relationship between socioeconomic conditions and the attitude of mothers in maintaining the dental health of stunting children.

**Materials and Methods** The research method uses correlational test with Spearman rank. The sampling technique used purposive sampling based on the inclusion criteria of mothers who had stunted children in Lengkong District, Bandung City.

**Result** Data analysis by the Spearman Rank resulted p-value of 0.019 ( *p* < 0.05) which leads to a positive relationship between socioeconomic conditions an mother's attitude in maintaining the dental health of stunting children.

**Conclusion** From the results of the study, it can be concluded that there is a relationship between socioeconomic conditions and the attitude of mothers in maintaining the dental health of stunting children in Lengkong District, Bandung City.

## Introduction

Stunting is a chronic malnutrition problem caused by the lack of nutritional consumption for a long time, causing children's growth to be lower than their age standards. The brain development of stunted children will be disrupted and affect cognitive abilities, productivity, and creativity at productive age. Stunting is a significant threat to the quality of Indonesian people to the nation's competitiveness. 1

The World Health Organization (WHO) limits the stunting problem in every country, province, and district by 20%. Meanwhile, several provinces in Indonesia still have high cases of around 30 to 40%. 2 According to the Bandung City Health Profile in 2019, Lengkong District had the highest stunting rate under 5 years at 14.35%. According to the Decree of the Mayor of Bandung, two subdistricts in Lengkong (Burangrang and Cikawao subdistricts) are the locus for a government program in stunting reduction and prevention. 3 At the beginning of 2022, there were around 50 children under 5 years with growth stunting in Lengkong District, Bandung.

Family income is related to the family's ability to fulfill primary, secondary, and tertiary necessities of life, including supporting dental health. Low income will affect the quality and quantity of food consumed by the family, such as a lack of protein, vitamins, and minerals, which function for children's growth, so that it can increase the risk of stunting. Research has shown a relationship between socioeconomic and maternal characteristics with the incidence of stunting in children under 5 years. 4

The oral health of growth-stunted children is more vulnerable than normal nutritional children. 5 6 In a study conducted by Hidayatullah et al in 2016 regarding the relationship between stunting and the level of dental caries, the caries index of stunting children was 8.23 with a very high category which was far from the caries index of normal nutrition children (3.3) currently. 7 Growth stunting conditions must be prevented to achieve a better next generation. Considerations to prevent stunting include improved nutrition, parenting, and education to change behaviors to improve maternal and infant nutritional health. Maintaining oral health in children with developmental delays requires interaction between children, parents, and dentists. 8 According to the Indonesian Dental Association, the role of parents is crucial in shaping a child's attitude, especially as the mother is the closest person to maintaining the child's oral health. Parents should pay attention to the health of their children's teeth, even if there are only deciduous teeth, because the condition of the deciduous teeth determines the future growth of permanent teeth. 6 Thus, this study aimed to analyze the relationship between socioeconomic conditions and mothers' attitudes toward maintaining oral health in children with growth stunting.

## Materials and Methods

This quantitative correlational study takes a population of mothers ( *n* = 35) who have children aged 6 months to 7 years in Lengkong District, Bandung. The sample size was total sampling, determined by purposive sampling with the inclusion criteria of having stunted children aged 6 months to 7 years. Stunting children who do not live with their mothers are excluded from this study.

### Instrument Development

The instrument in this study used a validated questionnaire containing questions regarding the parameters of socioeconomic conditions and maternal attitudes regarding the dental health of stunting children. The socioeconomic questionnaire contains 12 closed-ended questions covering age, gender, education level, occupation, and family income level and has been tested for reliability with Cronbach's *α* 0.750. 4 Meanwhile, the mother's attitude questionnaire was measured using a questionnaire with a Likert scale containing 12 questions and was given a score of 1 to 5 for each question. The questionnaire has been tested with a reliability value of Cronbach's *α* 0.924. 8 The questionnaire can be seen in Table 1 .

**Table 1:** The questionnaire used in the study

Socioeconomic questions	
1.	Father's last education level a. Elementary school b. Middle school c. High school d. Diploma e. University
2.	Mother's last education level a. Elementary school b. Middle school c. High school d. Diploma e. University
3.	Father's occupation a. Jobless b. Part time c. Full time
4.	Mother's occupation a. Jobless b. Part time c. Full time
5.	Father's income a. < 1,500,000 b. 1,500,000–2,500,000 c. 2,500,000–3,500,000 d. 3,500,000–5,000,000 e. > 5,000,000
6.	Mother's income a. < 1,500,000 b. 1,500,000–2,500,000 c. 2,500,000–3,500,000 d. 3,500,000–5,000,000 e. > 5,000,000
7.	Monthly family expenditure a. 354,000 b. 354,000–532,000 c. 532,000–1,200,000 d. 1,200,000–6,000,000 e. > 6,000,000
**Mother's attitude question, answered by selecting the appropriate number that resemble mothers attitude about oral health of their children (1) strongly disagree, (2) disagree, (3) neutral, (4) agree, (5) strongly agree**	
1.	Maintaining the oral health of toddlers by brushing their teeth twice a day is important
2.	Allowing children to drink bottled milk until they fall asleep contributes to cavities
3.	Tooth pain suffered by toddlers will affect their growth and development in the future
4.	Parents rarely give sweet food to their children for fear of cavities
5.	Parents take their children to the dentist from the first teething
6.	Parents routinely clean their child's teeth every day since he was a baby
7.	Parents limit their children to eat sweet and sticky snacks
8.	Letting children not chew their food will not affect the health of their teeth and mouth
9.	Toddlers don't have to be helped when they brush their teeth, because parents feel that their children are independent enough
10.	Parents don't force their children to refuse to brush their teeth, for fear of traumatizing them
11.	Parents have never taken their children to the dentist because they feel it's not the time yet
12.	Parents still give bottled milk as a lullaby for children

### Research Procedure

The research was conducted online from January to May 2022 due to the coronavirus disease 2019 (COVID-19) pandemic while still considering the respondent's rights and ethical values during its implementation. Mothers were gathered simultaneously through an online meeting platform and then instructed to complete a questionnaire. The participants were given Internet quota incentives for this study.

### Data Analysis

Research data analysis used the Spearman rank correlation test to determine the relationship between socioeconomic conditions and the mother's attitude in maintaining the oral health of growth stunting children.

## Result

The characteristics and family background of the participants can be seen in Table 2 . The characteristics of fathers who have stunting children in Lengkong District are high school graduates/equivalent (29; 82.86%). More than half of them work full time (19; 54.29%) with various incomes. In contrast, the mother's characteristics are mostly high school graduates/equivalent (24; 68.57%). Stay-home mothers were the majority (28; 80%) and did not have an income (26; 74.29%). Family expenditures are mostly 1,200,000 to 6,000,000 (15 families; 42.86%).

**Table 2:** Distribution of the characteristics of parents who have growth stunting children

Category	*n* (subjects)	%	Category	*n* (subjects)	%
Father education			Mother education		
Elementary school	1	2.86	Elementary school	3	8.57
Middle school	4	11.43	Middle school	8	22.86
High school	29	82.86	High School	24	68.57
Diploma	0	0.00	Diploma	0	0.00
University	1	2.86	University	0	0.00
Father occupation			Mother occupation		
Jobless	3	8.57	Jobless	28	80.00
Part time	13	37.14	Part time	6	17.14
Full time	19	54.29	Full time	1	2.86
Father's income			Mother's income		
< 1,500,000	8	22.86	< 1,500,000	26	74.29
1,500,000–2,500,000	9	25.71	1,500,000–2,500,000	5	14.29
2,500,000–3,500,000	9	25.71	2,500,000–3,500,000	3	8.57
3,500,000–5,000,000	9	25.71	3,500,000–5,000,000	1	2.86
> 5,000,000	0	0.00	> 5,000,000	0	0.00
Expenditure					
354,000	0	0.00			
354,000–532,000	3	8.57			
532,000–1,200,000	8	22.86			
1,200,000–6,000,000	15	42.86			
> 6,000,000	9	25.71			

The data in Table 2 are grouped into socioeconomic conditions of families with children with growth stunting, which is shown in Table 3 . It can be seen that the majority of families come from low socioeconomic conditions (68.57%). The mothers' attitudes regarding dental health are presented in Table 4 , which is still in a shallow stage.

**Table 3:** The socioeconomic conditions of families with stunted children

Social economy status	*n* (subjects)	(%)
Low	24	68.57
Middle	11	31.43
High	0	0.00
Total	35	100.00

**Table 4:** Mother's attitude about maintaining the dental health of stunting children

Mother's attitude	*n* (subjects)	(%)
Low	21	60.00
High	14	40.00
Total	35	100.00

The relationship between socioeconomic conditions and maternal attitudes regarding oral health shows the output value of Spearman's rho correlation of 0.394 with a significance value (two-tailed) of 0.019 ( *p* -value < 0.05). This shows a low relationship between socioeconomic conditions and the mother's attitude in maintaining the dental health of stunting children. With a positive correlation coefficient, the socioeconomic conditions were positively and significantly related to the mother's attitude ( Table 5 ). The higher the socioeconomic conditions, the higher the mother's attitude toward maintaining the dental health of stunting children.

**Table 5:** The relationship between socioeconomic conditions and the attitude of mothers in maintaining the dental health of stunting children

Spearman's rho correlation	Significant (two- tailed)	*N*
0.394 a	0.019	35

a Significant at level 0.05 (two-tailed).

## Discussion

Based on the research results above, most of the 35 respondents who have stunted children in Lengkong District, Bandung City, have low socioeconomic conditions. This socioeconomic condition is seen in the parents' last education level, parents' employment status, parents' income, and monthly family expenses. 4 Socioeconomic conditions determine the high and low position of a person's social position based on work to meet the needs of his life physically and spiritually. When economic conditions are not sufficient, social conditions will be disrupted. This is in line with the research conducted by Umar and Haryanto 9 in Indonesia and Mehta et al 10 in India, which shows that the socioeconomic conditions of the household simultaneously and partially influence stunting. Increased spending on food consumption can improve children's nutrition and nutrition to reduce stunting rates. Another study from Senegal stated that mothers' attitudes toward oral health and hygiene show the association between dental caries and oral hygiene index (OHI) level with other socioeconomic factors. 11 This supports the findings in this study, although the comparison of the number of subjects differs significantly.

It is generally agreed that mothers act as role models in shaping the oral health behavior of young children. 10 The mother's attitude in maintaining the dental health of stunting children in this study was mainly in the poor category. Attitude is a personality which determines actions and behavior toward an object accompanied by positive and negative feelings. A positive attitude will be formed if the stimulus gives a pleasant experience. Conversely, if the stimulus gives an unpleasant experience, a negative attitude will be formed. Attitude relates to the individual's readiness to react to particular objects according to the concept of positive–negative assessment. There are three related components in growing individual attitudes, namely cognitive, affective, and action tendencies. 12

From the data analysis, it is known that there is a significant and positive relationship between the socioeconomic conditions of the family and the mother's attitude toward maintaining their children's oral health. This correlation means the mother's attitude toward maintaining oral health will decrease when the family's socioeconomic conditions decline. Socioeconomic conditions are closely related to a person's health. In maintaining dental health, several health efforts refer to the WHO level of care, which consists of promotive, preventive, early detection, curative, and rehabilitative actions. 4 The use of these services will, of course, be different for each individual depending on a person's health behavior, namely predisposing factors (knowledge, belief values, behavior, and socioeconomics), supporting factors (health infrastructure and available drugs), and driving factors (family, friends, and other community leaders). 12

As the mother plays a vital role as the child's caretaker, the children's preventive practice of oral health tends to be controlled by their mother's attitude. A low level of awareness of oral health among mothers will ultimately reflect on the child's oral health and nutrition probelems. 13 14 15

### Study Limitation

Although due diligence was maintained to ensure the study's integrity, the findings are influenced by the limited sample size. Only mothers of growth stunting children from two districts were included in the study sample. Hence, the results, even though they may give insight into the current situation, may not be generalized to the whole population of mothers of a growth-stunting child in the Lengkong District of Bandung in 2022.

## Conclusion

Research findings regarding the relationship between socioeconomic conditions and maternal attitudes in maintaining the oral health of stunting children in Lengkong District, Bandung City, it can be concluded that there is a positive relationship between family socioeconomic conditions and mother's attitude toward maintaining the oral health of stunting children. The lower the socioeconomic condition of the family eating, the lower the mother's attitude toward maintaining the child's dental health. To get more accurate results, the authors recommend that further studies take primary data directly if the COVID-19 pandemic conditions have improved to cover more participants.
